# Whole Genome Analyses of the Endangered Northern Abalone (*Haliotis kamtschatkana*) Reveal Population Differentiation and a Genomic Signature of a Dramatic Population Decline

**DOI:** 10.1111/eva.70255

**Published:** 2026-06-04

**Authors:** Anna Tigano, Erin C. Herder, Kayla Long, Janine Supernault, Daniel L. Curtis, S. Christine Hansen, Mackenzie D. Mazur, Eric B. Rondeau, Dominique Bureau

**Affiliations:** ^1^ Pacific Biological Station, Fisheries and Oceans Canada Nanaimo British Columbia Canada

**Keywords:** connectivity, conservation genomics, inbreeding, mollusks, overfishing, population bottleneck

## Abstract

Despite widespread declines of many wildlife species, the effects of population decline on the genome and the recovery potential of affected species are still poorly understood, especially beyond a few charismatic species. The Northern abalone (or Pinto abalone; *Haliotis kamtschatkana* ) is a marine gastropod mollusk of social, cultural and historical economic importance in the Pacific Northwest of North America that experienced a decline in population density due to commercial harvest, and is currently listed as endangered in Canada under the Species at Risk Act. Previous genetic investigations based on microsatellites and reduced‐representation approaches concluded that Northern abalone is panmictic throughout its range, from Alaska to California, and identified high levels of genetic variation with no indication of population decline. Using whole genome resequencing data from Northern abalone sampled across the northern part of the species range, we instead identified both: (1) significant differentiation between two genetic groups, albeit concentrated in few genomic regions, and (2) a strong signature of a dramatic population decline, without genomic evidence of genetic inbreeding. Demographic reconstructions showed a modest signal of recent population expansion, supported by an increasing number of juveniles observed during dive surveys. We also found evidence of historical, rather than current, connectivity throughout the area investigated. These results are important for management decisions and highlight the utility of whole genome data in conservation, especially in species with historically large effective population sizes like the Northern abalone.

## 1 Introduction

Species losses and population declines are occurring at alarming rates and are mostly attributed to the direct and cascading effects of anthropogenic activities (Cowie et al. 2022). While severe population declines have been reported in thousands of species (WWF 2024), the effects of population bottlenecks on the genomes, and the ability to recover, of these declining species are not well understood and are hence difficult to predict. For example, the critically endangered vaquita porpoise ( *Phocoena sinus* ) has shown low inbreeding load and no signs of inbreeding depression, even though only ~10 individuals remain in the wild (Robinson et al. 2022). In contrast, the endangered Southern resident killer whale population ( *Orcinus orca* ), with ~70 individuals remaining (7× the vaquita census size), shows substantial inbreeding depression resulting in health issues that are preventing the population's recovery (Kardos et al. 2023).

The discovery of contrasting genomic signatures of population decline in these two relatively closely related species highlights the usefulness of whole genome data in making links between bottleneck and inbreeding, and characterizing extinction risks (Supple and Shapiro 2018; Taylor et al. 2021), among other things. In fact, the analysis of whole genome data enables the complete characterization of levels and distribution of genomic variation across the genome, which not only provides information on inbreeding, adaptive potential, and potential for population collapse or recovery, but also enables the reconstruction of demographic histories, which is crucial especially when baseline data are lacking. Together, this information is crucial to characterize the effect of population declines on the genome and inform the management and conservation of populations and species (Kardos et al. 2021; Hohenlohe et al. 2021). Additionally, whole genome analyses can reveal genomic differentiation that can potentially go undetected with the analysis of microsatellites or reduced‐representation approaches such as RAD‐seq (Atsawawaranunt et al. 2026). The increased resolution granted by the full coverage of the genome can result in a better understanding of population structure and its drivers (St. John et al. 2025) and in the identification of markers able to discern populations and species (Toews et al. 2016).

While a few vertebrate species have been intensely studied to investigate the genomic signature of their decline (e.g., Kardos et al. 2023; Khan et al. 2021; Robinson et al. 2022), invertebrates—representing 95% of animal biodiversity—are heavily underrepresented in conservation genomics research (Lopez et al. 2019). For example, many abalone species, marine gastropod mollusks known for their economic importance as a seafood delicacy, have suffered population declines driven by overharvesting and illegal harvest (Cook and Shumway 2023), and some species have suffered high mortality from diseases (Rogers‐Bennett et al. 2002); yet, the genomic consequences of these declines have rarely been investigated (but see Wooldridge, Orland, et al. 2024; Wooldridge, Kapp, et al. 2025). The Northern abalone (or Pinto abalone; *Haliotis kamtschatkana* ) experienced a dramatic population decline across its range, spanning from Southeast (SE) Alaska (USA) to Baja California (Mexico), and is currently listed as Endangered in Canada (Species at Risk Act (SARA) Species Registry) and on the International Union for Conservation of Nature (IUCN) Red List (Peters and Rogers‐Bennett 2021). Overharvesting was the major driver of population decline in Canada and all commercial, recreational and First Nations' food, social and ceremonial fisheries were closed in 1990 (Campbell 2000), though illegal harvesting continues to be a threat for the species (Neuman et al. 2018).

Despite the well‐documented decline of Northern abalone, previous studies based on 12 microsatellites (Withler et al. 2003) and ~6000 SNPs (Dimond et al. 2024) found overall high levels of genetic variation, and estimated large effective population sizes with no signature of population bottleneck. Northern abalone displays a patchy distribution (spanning over 3700 km and 30 degrees of latitude), and life history traits, including a relatively short planktonic larval phase (7–10 days; Strathmann 1987) and low adult dispersal (Sloan and Breen 1988), that would suggest population differentiation across its range. However, neither study identified significant population structure and differentiation across the species range, and concordantly concluded that the species is panmictic from Alaska to Mexico (Withler et al. 2003; Dimond et al. 2024). Lack of population structure was attributed to levels of current or historical gene flow sufficient to homogenize genetic variation across a large geographical range. As a result, in Canada, Northern abalone is managed on a coastwide basis as a single Designatable Unit (DU; COSEWIC 2009). Even if marine species are often associated with weak to no population structure, promoted by an apparent lack of barriers to gene flow in the ocean, marine invertebrates with longer planktonic phases, such as the bat star ( *Patiria miniata* ; 6–10 weeks) and the giant red sea cucumber ( *Apostichopus californicus* ; 2–4 months) have shown significant population structure within similar ranges as the Northern abalone, even with fewer markers (7 microsatellites and ~3000 SNPs, respectively; Sunday et al. 2014; Xuereb et al. 2018). Furthermore, in high gene flow scenarios differentiation, if any, tends to concentrate in the genome (Yeaman 2013), and is hence easy to miss with reduced‐representation data (e.g., Luna et al. 2023). It is therefore plausible that the genetic markers previously used to investigate population structure and demographic trends of Northern abalone were not sufficient to identify signatures of differentiation and population decline due to limited resolution, high levels of genetic variation, and/or concentrated genomic signatures of bottleneck and differentiation.

Here, we used whole genome data from 15 locations across the northern part of the Northern abalone range (BC and SE Alaska) to: (1) characterize population structure and differentiation, if any; and (2) determine the effect of the severe species decline in the genome. The results from this work provide important information for the management of Northern abalone in Canada and a better understanding of the effect of population declines in understudied taxa such as mollusks.

## 2 Methods

### 2.1 Library Preparation and Sequencing

**FIGURE 1:**
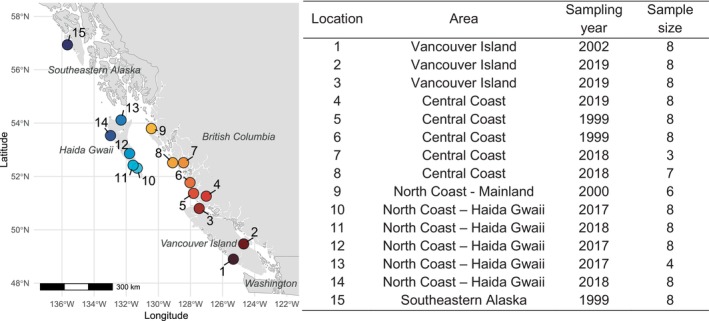
Map showing sampling locations and table summarizing area and sample size information. Coordinates and precise locations are restricted for the endangered Northern abalone under Section 124 of the Species at Risk Act.

We used epipodial tissue samples that were collected non‐destructively between 1999 and 2019 from 111 individuals from 15 sampling locations; 14 locations along the BC coast and one location in SE Alaska, as per the methods described in Withler et al. (2003) and a series of SARA collection permits for the work (Figure 1). We extracted DNA using a Qiagen Biosprint 96 DNA extraction kit. Whole genome library preparation and sequencing was performed at Genome Québec. Genomic DNA was quantified using the Quant‐iT PicoGreen dsDNA Assay Kit (Life Technologies). Libraries were generated from 100 ng of DNA using the NEBNext Ultra II DNA Library Prep Kit for Illumina (New England BioLabs) as per the manufacturer's recommendations. Size selection of libraries was performed using SparQ beads (Qiagen). Libraries were quantified using the KAPA Library Quantification Kits—Complete kit (Universal; Kapa Biosystems) and their average fragment size was determined using a Fragment Analyzer 5300 (Agilent) instrument. The libraries were normalized, pooled, and sequenced on an Illumina NovaSeq X platform using 150 bp paired‐end reads.

### 2.2 Variant Calling

We used the snpArcher pipeline for variant calling (Mirchandani et al. 2024). SnpArcher takes raw sequencing data, processes them, and calls sequence variants from whole genome resequencing data. We used GATK (McKenna et al. 2010; Van der Auwera et al. 2013) as the variant caller, with all default settings other than selecting the low coverage option. We ran the pipeline twice using two different heterospecific reference genomes for read mapping as a high‐quality reference genome for the Northern abalone was not available at the time of this study. We used the black abalone ( *Haliotis cracherodii* ) reference genome as it is the most contiguous abalone reference genome available, and the red abalone ( *H. rufescens* ) reference genome, as, with white abalone ( *H. sorenseni* ), is the Northern abalone's closest relative, and divergence between the two species is shallow (Crosson and Friedman 2018; Masonbrink et al. 2019), which should minimize reference bias (Akopyan et al. 2025). The proportion of mapped reads (98.6% vs. 99.1%), the proportion of properly paired reads (87.7% vs. 91.7%), and the number of high‐quality SNPs (47 million SNPs vs. 56 million SNPs, see Section 3) were higher when the data were mapped to the red abalone genome, the less divergent reference genome, compared to the black abalone genome, the most contiguous reference genome, so we retained the red abalone reference genome for subsequent analyses. Note that we renamed reference genome scaffolds with numbers in order from largest to smallest.

We applied additional quality filters to the raw variants output by snpArcher with VCFtools v0.1.16 (Danecek et al. 2011). We filtered out variants with depth < 4 and > 30 (1/3 and 3 times the average coverage, respectively) and with more than 15% missing data, kept only biallelic sites and removed indels (“full dataset”). We then split the variants from the full dataset into two additional datasets based on their minor allele frequency (MAF) calculated across all individuals: (1) the ‘common alleles’ dataset included variants with MAF ≥ 0.05 and (2) the ‘rare alleles’ dataset including variants with MAF < 0.05. The three datasets (full, common, and rare) were used for different analyses downstream: the full dataset was used for unbiased measures of genetic diversity and demographic reconstructions, the common allele dataset was used for estimates of genomic differentiation between locations and groups, and the rare alleles dataset was used to test hypotheses about population connectivity and demography.

Preliminary analyses, including a Principal Component Analysis (PCA), and coverage analysis, identified two individuals showing anomalous divergence from the rest of individuals probably due to sample contamination, and one individual with more than 15% missing data due to low genome coverage. These three individuals were excluded from downstream analyses (*n* = 108).

### 2.3 Population Structure and Patterns of Genomic Differentiation

We explored population structure using a PCA with the package SNPRelate (Zheng et al. 2012) in R 4.4.1 (R Core Team 2026). We first used a random subset of 100,000 common allele SNPs to conduct an exploratory PCA. We then tested for the minimum number of SNPs necessary to retrieve a signal of population structure by performing PCAs with 10,000, 1000, 100 and 10 MAF‐filtered SNPs. We also ran a PCA with 1 million SNPs to test whether increasing the number of SNPs would reveal finer population structure.

From these analyses we identified two genetic groups: a southeast group, including locations 1–9, and a northwest group, including locations 10–15 (see Section 3). To characterize patterns of differentiation in the genome and identify differentiated loci between these two genetic groups, we estimated *F*_ST_ at each individual SNP and in 50‐kb moving windows using VCFtools. Then, we selected the most differentiated SNPs, that is, the SNPs showing the highest *F*_ST_ between the southeast and the northwest groups, created subsets including the 10, 100, 1000, 10,000, 100,000 and 1 million most differentiated SNPs respectively, and performed PCAs using each of these sets of SNPs.

We further examined the 1000 most differentiated SNPs in the genome. We calculated their MAF in each location and across groups, and their difference in allele frequencies (ΔAF) between the northwest and the southeast groups. Furthermore, we considered these 1000 SNPs for further functional exploration. We functionally annotated the SNPs including the genes that were directly affected (i.e., the SNP that fell on the gene sequence) and the genes that were closest to SNPs falling in intergenic areas (based on the assumption that SNPs in intergenic areas, potentially regulatory, would affect the closest gene in the linear genome) using BEDtools functions in the *valr* v0.8.4 package in R (Quinlan and Hall 2010; Riemondy et al. 2017). We used various databases, including SwissProt, NCBI, and GeneCards, (Boutet et al. 2007; NCBI 1988; Stelzer et al. 2016) to gather information on the function of genes associated with differentiated variants.

### 2.4 Genomic Diversity

To assess levels and distribution of sequence variation among samples and across the genome, we calculated nucleotide diversity and Tajima's *D* in 50‐kb moving windows using the full dataset across all individuals combined, and separately across each of the two identified genetic groups (northwestern and the southeastern groups, see Section 3). We also calculated individual heterozygosity using the full and the common alleles dataset. All analyses were performed in VCFtools.

### 2.5 Demographic Reconstructions

We reconstructed the demographic history of Northern abalone with GONE (Santiago et al. 2020), a software that uses patterns of Linkage Disequilibrium (LD) between SNPs to estimate changes in effective population size (*N*_e_) up to 140 generations in the past, where generation is defined as the average age of reproducing individuals in a population. We set a maximum of 50,000 SNPs per chromosome, the Haldane correction for genetic distance, and 40 replicates. As recombination rates in mollusks vary broadly (Stapley et al. 2017), we ran the demographic reconstructions using three recombination rate estimates representing the average recombination rate, 1 cm/Mb, and approximately the extremes of the mollusks' recombination rate variation, 0.5 and 2 cM/Mb, with the rest of the parameters kept the same, which enabled us to quantify the effect of varying recombination rate on the timing of demographic events and *N*_e_ estimates in the demographic reconstructions.

To generate the input files for these analyses, we thinned the full dataset, including all individuals, only northwest individuals, or only southeast individuals, respectively, and retained only SNPs that were located farther than 1 kb from each other and on scaffolds longer than 10 Mb, and then further randomly selected 500,000 SNPs.

### 2.6 Runs of Homozygosity

To evaluate whether the recent population decline of Northern abalone has led to genetic inbreeding, we calculated Runs of Homozygosity (ROHs) in each individual using Plink v1.9 (Clarke et al. 2011) setting minimum ROH length to 100 kb. ROHs are long stretches of identical‐by‐descent (IBD) haplotypes that appear in individual genomes as the result of recent parental relatedness, and as such are unlikely to occur independently in the same position in different locations. We collapsed ROH segments identified across multiple individuals into unique ROHs if they overlapped by more than 50% and evaluated how many individuals and locations shared the same ROH segments. Based on their low homoplasy, we calculated the number of individuals sharing ROHs between locations with the *crossprod* function in R as an indicator of connectivity among sampling locations. As ROHs shorten with time due to recombination with non‐homozygous genomic regions, we can estimate ROHs coalescent times from their lengths (Foote et al. 2021; Thompson 2013) and infer historical demographic events at varying points in time, including bottlenecks and historical connectivity. To estimate the age of ROHs in number of generations, we used the formula *t* = [100/(2 × L × *r*)] cM where *t* is coalescent time in number of generations, *L* is length in Mb, and *r* is recombination rate (Foote et al. 2021; Thompson 2013). In the absence of recombination rate estimates for abalone, we used the average recombination rate estimated for mollusks (1 cM/Mb; Stapley et al. 2017).

### 2.7 Analysis of Rare Alleles

As an additional measure of connectivity and signature of population contraction and expansion, we examined the distribution of rare alleles within individuals and locations. We calculated the number of singletons and doubletons, that is, the number of variants present in only one individual in either heterozygous or homozygous state (i.e., one or both alleles), and the number of locations each rare variant was shared across using VCFtools and R.

## 3 Results

### 3.1 Genomic Diversity

We identified a total of 56.4 million high quality SNPs (full dataset), indicating, with 1 SNP every ~24 bp on average, high overall genomic diversity. Of these SNPs, only 19.1 million (34%) had MAF > 0.05 (common alleles dataset). After quality filtering (including for MAF), high quality SNPs had an average sequencing depth of 9.7× (SD = 1.3×) and 9% (SD = 3.6%) missing data.

Mean heterozygosity, that is, the proportion of heterozygous sites in each individual, was 11.5% in the full dataset and 28.8% in the common allele dataset, with significant differences only between location 12, showing highest overall heterozygosity, and five other locations (1, 3, 4, 11, and 15), showing the lowest mean heterozygosity (Tukey Post Hoc test, *p* < 0.05). Mean nucleotide diversity across 50‐kb windows was 4.17 × 10^−3^ across all individuals (*n* = 108), 4.14 × 10^−3^ in the northwest (*n* = 44), and 4.18 × 10^−3^ in the southeast (*n* = 64). Mean Tajima's *D* across all genomic 50‐kb windows was negative across all individuals (Tajima's *D* = −1.04), and within the southeast (Tajima's *D* = −0.93) and the northwest group (Tajima's *D* = −0.90; Figure S1), indicating an overall excess of rare alleles.

### 3.2 Population Structure and Genomic Differentiation

**FIGURE 2:**
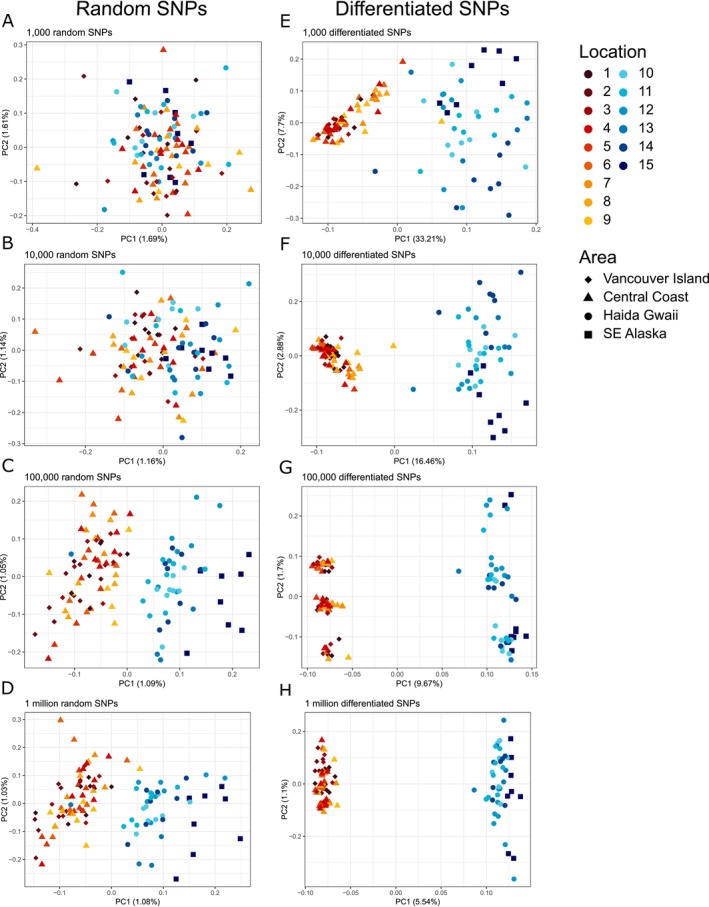
Plots of Principal Component Analyses based on an increasing number of random SNPs (A–D) and differentiated SNPs (E–H) from 1000 on the top to 1 million at the bottom.

The PCA based on 100,000 random SNPs revealed differentiation between two geographic groups: (1) Vancouver Island and the mainland coast of BC (locations 1–9), hereafter referred to as “Southeast group,” and (2) Haida Gwaii and SE Alaska (locations 10–15) termed the “Northwest group” (Figure 2C). Individuals were separated mostly along PC1, except one individual from site 13 in the Northwest group, clustering with individuals from the Southeast group. The PCAs based on less than 100,000 random SNPs showed decreasing group definition: with 10,000 SNPs, individuals tended to cluster within each of the two groups but did not form clearly distinct groups anymore, and with smaller datasets, individuals lost any population structure signal (Figure 2A,B). The PCA based on 1 million SNPs showed a similar clustering pattern as with 100,000 SNPs and did not reveal finer population structure (locations 10–14; Figure 2D).

**FIGURE 3:**
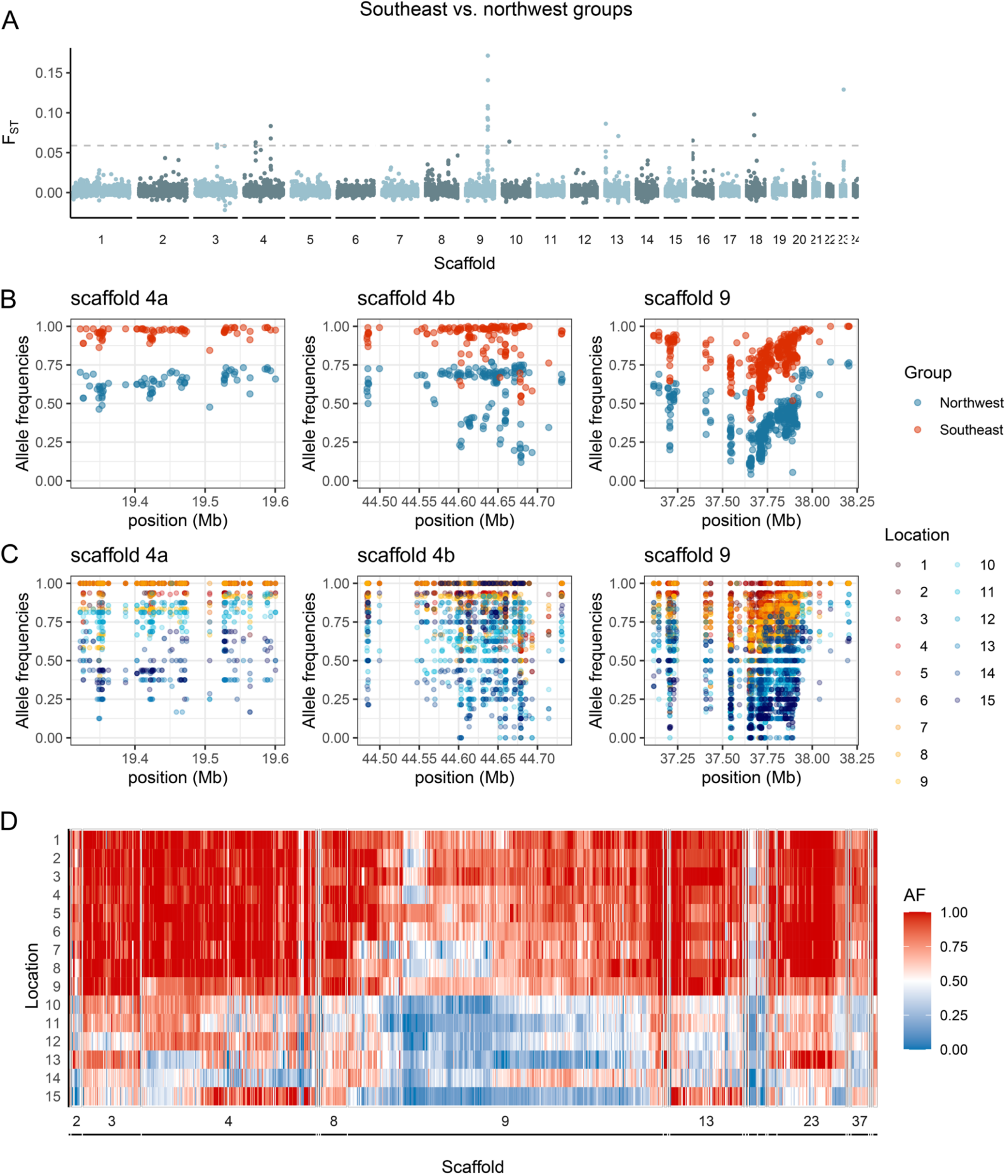
Patterns of genomic differentiation between the northwest and southeast groups. (A) Manhattan plot showing patterns of differentiation calculated as *F*_ST_ in 50 kb moving windows across the genome. (B, C) Plots showing variation in allele frequencies at each of the 1000 most differentiated SNPs located along the three main AODs on scaffold 4 (4a and 4b) and 9 calculated within genetic groups (B) and individual locations (C). (D) Genotype plot summarizing allele frequency at each of the 1000 most differentiated SNPs across all scaffolds. The differentiated SNPs were located across 24 scaffolds, but only the eight scaffolds with the highest number of differentiated SNPs were labeled in the plot.

The genomic landscape of differentiation between the two genetic groups showed overall low background *F*_ST_ (average *F*_ST_ across SNPs = 0.001146 and across windows = 0.001386) with a few 50‐kb windows showing higher differentiation (Figure 3A). Genomic differentiation tended to be concentrated in a few regions of the genome: of the 27 windows with *F*_ST_ values above the 99.9th percentile (“outlier windows”), eight were found on scaffold 9 and five on scaffold 4. Excluding three short scaffolds (< 125 kb) where outlier windows were likely artifacts, only seven additional scaffolds (for a total of nine) harbored outlier windows. Similarly, at the SNP level, of the 1000 most differentiated SNPs, 404 were located on scaffold 9 and 223 were located on scaffold 4 (Figure 3B,C). The remainder were distributed across 22 additional scaffolds. However, only 12 of these scaffolds included more than 10 highly differentiated SNPs.

PCAs based on highly differentiated SNPs revealed a clear signature of population structure (Figure 2E–H), even with as little as the 10 most differentiated SNPs (Figure S2), with distance between the two groups increasing slightly with increasing number of SNPs (Figure 2G,H). None of the 1000 most differentiated SNPs were fixed between the northwest and the southeast groups (Figure 3B–D), but 65 SNPs were fixed in the southeast group and variable in the northwest. The two northernmost locations, 13 and 15, shared 54 and 81 fixed differences with locations from the southeast group, 50–81 more than the other northwest locations. Note that the fixed SNPs shared with the southeast were located in different parts of the genome in the two locations: those fixed in location 13 were mostly concentrated on scaffold 23, while those fixed in location 15 were mostly concentrated on scaffold 4 and 13, with no shared fixed differences between locations 13 and 15.

We identified 91 genes associated with the 1000 most differentiated SNPs, 26 of which were uncharacterized (Table S1). The two areas of differentiation (AOD) on scaffold 4 were associated with four and six genes, respectively. In the first AOD, only one gene was characterized, a zinc finger protein gene, while on the second AOD, the six annotated genes were mostly associated with immune and reproductive functions. The area of differentiation on scaffold 9 was associated with 14 genes, and eight of these were functionally annotated with functions mostly associated with immune functions, stress response, and apoptosis (Table S1).

### 3.3 Effective Population Size and Population Demography

**FIGURE 4:**
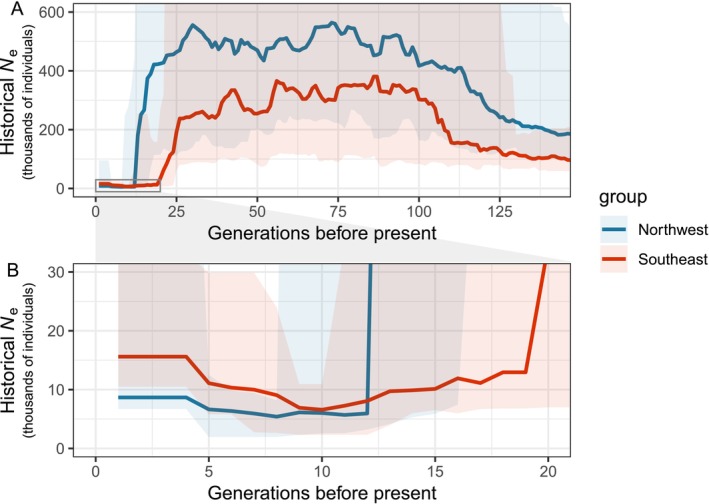
Plots summarizing the results from the demographic reconstructions from GONE (A) for the last 140 generations and (B) a zoom‐in on the last 20 generations before sampling began in 1999. The thick solid line represents the median across 40 iterations and the shaded area the 95% confidence intervals.

To reconstruct the recent demographic history of Northern abalone in BC and SE Alaska we used multiple independent sources of information: (1) LD, (2) Tajima's *D*, and (3) analysis of rare alleles. We first reconstructed the demographic history over time using genomic patterns of LD among SNPs. Using three different recombination rate values (0.5, 1, and 2 cM/Mb) for the demographic reconstructions showed an inverse relationship between recombination rate and both *N*_e_ and time, that is, with increasing recombination rates both estimates of *N*_e_ and number of generations before presents of demographic events decrease; however the population decline pattern, and the differences between the two genetic groups were evident across the different recombination rates (Figure S3). This said, here we present in more detail the results on reconstructions based on the average mollusk recombination rate of 1 cM/Mb (Figure 4). Between 140 and 100 generations before present, both southeast and northwest groups appeared to have increased 2–3 fold their effective population size (*N*_e_) in 30–35 generations, which was followed by a long period of relative population stability. More recently, large declines in *N*_e_ across BC and SE Alaska were observed. The northwest and southeast groups both showed a severe bottleneck, but differed in the timing of the population decline and their historical effective population sizes. Before the bottleneck, *N*_e_ was relatively stable around 300,000 individuals in the southeast group, and around 500,000 in the northwest group. Effective population sizes started slowly declining in both groups approximately 39 generations before present, but the population bottlenecks occurred earlier in the southeast group. Northern abalone declined by more than 50% (compared to generation 39) at generation 25 before present in the southeast group and at generation 16 before present in the northwest group. From this point, *N*_e_ in both groups further decreased to 10% in only 3–4 generations. *N*_e_ reached its historical minimum 8–10 generations before present with a decline to 0.76% and 1.8% of their pre‐bottleneck average in the northwest and in the southeast groups, respectively.

GONE also detected a signature of recent population expansion in the last few generations, showing that *N*_e_ almost tripled since the lowest estimate was reached approximately 8–10 generations before present in both groups. However, current *N*_e_ estimates were only 5% and 2% of the average estimates from the 100 generations prior the bottleneck, in the southeast and northwest groups respectively.

Estimates of Tajima's *D* were negative across most of the genome, indicating an overall excess of rare alleles (Figure S1), which, being so pervasive across the genome, was indicative of population expansion rather than selection. Rare variants, those with overall minor allele frequency < 0.05, were abundant, making up 66% of all variants. The high proportion of rare variants found in only one individual or location (42% and 28% of all variants) further suggests that a large number of new mutations arose recently, more so in the northwest group than the southeast group, based on the higher number of singletons in locations from the northwest (Figure S4). These data indicate that these rare variants have not had the time to increase in frequency and to spread within and among locations, further supporting that the Northern abalone is slowly recovering from a relatively recent dramatic population bottleneck.

### 3.4 ROHs Calling

**FIGURE 5:**
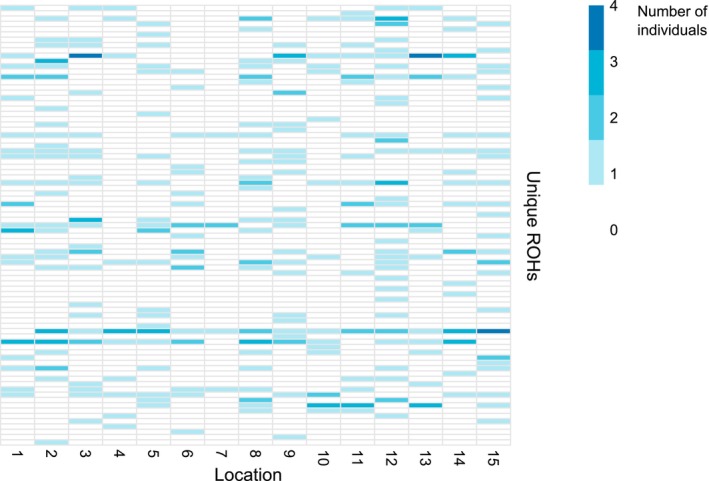
Distribution of ROHs across locations where each row represents a unique ROH, and the color intensity represents how many individuals in a given location carry that unique ROH.

We identified a total of 83 ROH segments across 108 individuals. On average, individuals carried 3.4 ROHs (number of ROHs, NROH), covering 647 kb (sum of ROH lengths, SROH), or 0.05% of their total genome length, and ROH segments were 193 kb long on average. *F*_ROH_, a measure of inbreeding, is generally reported as the proportion of the genome affected by ROHs longer than 1.5 Mb, as a link has been suggested between ROHs of this length and inbreeding (McQuillan et al. 2008). In our dataset, the longest segment was 1.05 Mb, the only one above 1 Mb, indicating no detectable inbreeding. We did not observe any geographical patterns in the distribution of ROHs across the range (Figure 5), and both NROH and SROH did not differ significantly among locations, groups (southeast vs. northwest), or areas (Vancouver Island, mainland coast of BC, Haida Gwaii, and SE Alaska; ANOVA, *p* > 0.05; Figure S5). Furthermore, ROH sharing across locations did not show a geographical pattern either (Figure 5, Figure S6). Estimated coalescent times of ROHs ranged from 48 to 500 generations before present. The two most widely shared ROHs had coalescent times of ~400 and 227 generations before present.

The analysis of rare alleles revealed that 15,761,026 variants (28% of all variants and 42% of rare variants) were private, that is, present in one location only, and the vast majority of these (15,189,795 variants, 26% of all variants and 41% of rare variants) were present in only one individual, mostly in a heterozygous state (96.2%). These results highlight that a large portion of the total genomic variation in Northern abalone is made up of very rare alleles, that probably arose recently as new mutations following the bottleneck.

## 4 Discussion

Using whole genome data and multiple analytical approaches, we have addressed fundamental questions related to the population structure and the demographic decline observed in Northern abalone from BC and SE Alaska, which will help inform their management in Canada and beyond. Our study shows that in Northern abalone, a species with high levels of genomic variation and weak differentiation, whole genome data are necessary to characterize population differentiation, genomic variation and demographic histories, and that reduced‐representation approaches, including microsatellites, have failed to capture genomic signatures that may be relevant for management and conservation. While reduced‐representation approaches present some advantages compared to whole genome sequencing, for example, they are generally cheaper and require fewer genomics and bioinformatics resources, they can miss important information or even be misleading (Atsawawaranunt et al. 2026). In the case of Northern abalone, a complete characterization of genomic differentiation could only be obtained by sampling the entirety of the genome.

We have also shown how informative data that are generally discarded can be, such as with rare alleles, or when used for a single specific application, such as ROHs. In this study, rare alleles were instrumental to better reconstruct population trends and characterize signals of population recovery and connectivity. Similarly, ROHs were used not only as indicators of current genetic inbreeding, but also to infer historical inbreeding and connectivity among distant sampling locations.

### 4.1 Genomic Differentiation in Northern Abalone Is Concentrated and Driven by Selection and Historical Connectivity

Our analyses have corroborated previous genetic work reporting weak genome‐wide differentiation (Withler et al. 2003; Dimond et al. 2024), but have also identified two distinguishable genetic groups, one including locations from Vancouver Island and the mainland coast of BC, and another including locations from Haida Gwaii and SE Alaska. Genomic differentiation was very concentrated in the genome, mostly on two scaffolds, 4 and 9, which explains why it had not been identified previously. In fact, we determined that a number of randomly sampled SNPs in the hundreds of thousands order of magnitude is required to detect population differentiation in Northern abalone, and that the population differentiation signal is quickly lost in analyses based on a lower number of random SNPs. In contrast, due to the concentrated nature of the genomic differentiation, a low number of highly differentiated SNPs, as little as 10, was sufficient to distinguish the two groups. Although low adult dispersal capabilities and relatively short pelagic larval phases would suggest stronger population structure than detected, in historically large populations like in the Northern abalone (*N*_e_ = 300,000–500,000, assuming an average mollusk recombination rate), genetic drift is weak and even minimal gene flow can further hamper differentiation (Slatkin 1993). The combination of historically large population size, selection, weak genetic drift, and gene flow (seemingly more historical than current) is likely what promotes the concentration of genomic differentiation in Northern abalone (Tigano and Friesen 2016). Structural variants also likely enable the maintenance of these genomic patterns (Mérot et al. 2020), and their role in differentiation and local adaptation in Northern abalone is an open question that will be best addressed when a species‐specific high quality reference genome becomes available.

These results generally align with other historically abundant abalone species, which tend to display overall weak to no population structure (e.g., red abalone; Griffiths et al. 2025), with a few differentiated markers associated with latitudinal gradients (e.g., black abalone; Wooldridge, Orland, et al. 2024), or environmental variation (e.g., red abalone and greenlip abalone; De Wit and Palumbi 2013; Sandoval‐Castillo et al. 2018).

In Northern abalone, the strongest signals of differentiation were mostly associated with genes involved in immune and stress response. Abalone worldwide are susceptible to disease. Withering syndrome, for example, almost exterminated black abalone throughout their range in California and Mexico (Rogers‐Bennett et al. 2002). In laboratory exposure experiments, Northern abalone was the most susceptible to withering syndrome of all the Northeastern Pacific abalone species (Crosson and Friedman 2018). Yet, diseases, including withering syndrome, have never been reported or tested as a major cause of population decline for Northern abalone; however the two genetic groups may have been impacted differently by diseases in the past, leaving a stronger signature of selection, that is, lower variation, in the southeast group. Two additional differentiated genes linked to reproductive functions (oocyte development [*PAQR5*] and sperm development [*SPAG16*]) curiously co‐occurred in the same area of differentiation on scaffold 4. While these genes might hint at the evolution of reproductive isolation between the two genetic groups, this link is speculative in the absence of more information on phenotypic differentiation. Immune and reproductive function genes were shown to be under selection also in black abalone (Wooldridge, Kapp, et al. 2025), decimated by withering syndrome in the late 1900s, supporting the hypotheses that diseases may have similarly left a signature of selection in Northern abalone genomes. Nonetheless, the stark contrast between negligible genome‐wide differentiation and concentrated areas of differentiation in Northern abalone suggests that either selection in these areas is strong, or that alternative alleles evolved in isolation, and were maintained by some recombination‐suppression mechanism or selection against recombinants upon secondary contact (Guerrero and Hahn 2017; Han et al. 2017; Nelson and Cresko 2018).

Overall, the geographic patterns of population differentiation did not adhere to an isolation‐by‐distance model nor to expectations based on major oceanic current circulation in coastal BC, such as in the giant red sea cucumber, where population structure is consistent with the North Pacific Ocean current split into the Alaska Current northward and the California current southward off the coast of Vancouver Island (Xuereb et al. 2018). However, the population structure of the bat star is similar to the Northern abalone's, with individuals from Haida Gwaii and SE Alaska forming a distinct group from the rest of BC. Ocean circulation models of larval dispersal in the bat star showed how individuals from Vancouver Island were potentially connected with BC Central and North Coasts, and how isolated individuals from Alexander Archipelago in SE Alaska and, to a greater extent, Haida Gwaii were from the rest of the BC coast (Sunday et al. 2014). The different patterns of connectivity along the BC coast in different marine species may be explained by their different larval life histories, spawning bahavior, settlement cues, and habitat requirements. Species with longer pelagic larval phases are likely to be affected by broader current circulation and transported over longer distances than species with shorter larval phases, which are expected to be more strongly affected by finer‐scale circulation patterns. However, pelagic larval duration may not necessarily be a good predictor of genetic distance (Esser et al. 2023), and other factors, unrelated to larval dispersal bahavior, such as deeper evolutionary histories, may leave a stronger signature than patterns of larval dispersal across a species range.

The greater historical *N*_e_ and higher variation in the highly differentiated SNPs in the northwest group compared to the southeast group, together with the greatest mean individual heterozygosity in the Haida Gwaii region, is consistent with the hypothesis that Haida Gwaii, and potentially the Alexander Archipelago, may have been glacial refugia during the Last Glacial Maximum for many marine and terrestrial species (Shafer et al. 2010; Pruett et al. 2013; Wu et al. 2022; Mathewes and Clague 2017), including the Northern abalone. Furthermore, the sharing of fixed differences between the southeast group and the two northernmost locations on Haida Gwaii and SE Alaska further supports that these two northernmost locations may have been historically connected with Northern abalone from the mainland coast of BC and/or represent areas of secondary contact following the Last Glacial Maximum, but independent in space or time from each other. Taken together, these results indicate that genomic differentiation in the northern range of the Northern abalone is primarily driven by historical segregation and selection in a few concentrated areas of the genome.

### 4.2 Genomic Analyses Reveal a Strong Genomic Signature of Population Decline but No Inbreeding

Our reconstructions based on three different recombination rates show similar population trends, but illustrate also how varying recombination affects dating of demographic events and *N*_e_ estimates. While here we discuss the results based on the recombination rate 1 cM/Mb, at this time it is not possible to temporally calibrate our demographic reconstructions due to not only unknown recombination rates in Northern abalone, but also to uncertainty associated with generation time, which may vary between 2 and 10 years and be affected by variation in sea otter predation, food availability, and growth rates across the species range (Obradovich et al. 2021), thus adding another layer of uncertainty in the dating of demographic events.

Despite high overall genomic variation, we identified bottlenecks in both genetic groups resulting in 98%–99% effective population reductions, with steep declines over approximately six generations that were not detected in previous genetic analyses (Withler et al. 2003; Dimond et al. 2024). The population trends reconstructed here from genomic data are consistent with the evidence of the severe decline documented during dive surveys in the period 1978–2007, showing a reduction of 88%–89% of mature individuals across BC and up to 98% in the Strait of Georgia (COSEWIC 2009). Other sources of knowledge, including zooarcheological, historical, traditional and western science evidence (Lee et al. 2019), provide information further back in time and together with survey data support that Northern abalone had large effective population sizes and that in only a few generations numbers plummeted dramatically. Our analyses hint at the start of a modest recovery in the last 8–10 generations, possibly a result of the closure of all Northern abalone harvest in BC in 1990. The signal of recovery from the demographic reconstructions, based on patterns of LD, is supported by a pervasive excess of rare alleles across the genome, which cannot be attributed to selection but rather indicate population expansion. Further, the high proportion of singletons/doubletons—more than 1 in 4—indicate that these alleles are likely new mutations that have arisen recently and have not had the time to increase in frequency by drift or selection. The abundance of these rare alleles was thought to be the result of high mutation rates in abalone species (De Wit and Palumbi 2013), but a recent study shows that white abalone ( *Haliotis sorenseni* ) has mutation rates comparable to vertebrates with similar longevity and generation times (Wooldridge, Ford, et al. 2025), suggesting that population recovery is occurring over longer timescales. Indications of population recovery from the genomic data are supported by empirical observations of increasing densities of Northern abalone, though mostly of small juveniles (Lee et al. 2019; Obradovich et al. 2021). These results highlight how informative rare variants, which are generally discarded from population genomics analyses, can be about recent demography. Had we not considered the allele frequency of the numerous SNPs identified or run demographic reconstructions, the amount of variation alone would not have indicated population declines or recent recovery.

Despite the dramatic population decline, we did not identify an amount nor size distribution of ROHs indicative of inbreeding. In human populations inbreeding seems to be more strongly associated with small population size rather than consanguinity (Ceballos et al. 2021), therefore, the large historical population sizes and existing high genomic variation before the bottleneck may have prevented inbreeding following the population collapse. Alternatively, either the signature of inbreeding is lagging due to maintained genomic variation, or the abundance of new mutations that occurred after the bottleneck may have masked the lack of genomic variation that characterizes ROHs.

Using a heterospecific reference genome for read mapping may cause reference bias and underestimation of ROHs sizes and numbers, with bias increasing with sequence and structural divergence (Prasad et al. 2022; Akopyan et al. 2025). Red abalone, used as a reference genome, and Northern abalone are closely related (Crosson and Friedman 2018; Masonbrink et al. 2019), as further corroborated by high mapping rates in our study. Although reference bias is expected to be minimal based on the available information, it cannot be quantified without a high‐quality Northern abalone reference genome. Nonetheless, low genomic differentiation, high levels of genomic variation, and historical large *N*_e_ together support lack of detectable inbreeding. In the future, a high‐quality Northern abalone reference genome will enable comparative genomics analyses and an additional validation of these results.

ROHs are identical by descent segments and as such can be used as genetic markers to infer not only inbreeding, but also recent demography and connectivity. One advantage of ROHs over SNPs is that they are less susceptible to homoplasy, i.e., the same variant arises independently in different individuals and locations. As such, they are more reliable to infer connectivity. Homoplasy would be an extremely unlikely explanation for the sharing of one or both breakpoints of hundreds‐of‐kb‐long ROHs, often of the same lengths, across individuals. In our dataset, sharing of ROHs across distant sampling locations suggests that periods of localized inbreeding in the past were followed by dispersal and gene flow, probably associated with range expansions and contractions, which carried those ROHs across long distances. During the Holocene only, the coast of BC and SE Alaska was highly dynamic, with sea levels much higher or lower than today, and changing rapidly (Josenhans et al. 1995). Changes in coastline, exposed land, and currents might have increased or decreased connectivity over time across the Northern abalone range, potentially explaining the spatial distribution of ROHs and blocks of differentiation in BC and SE Alaska.

### 4.3 Management Implications

Northern abalone is managed as a single Designatable Unit in Canada (Species at Risk Public Registry 2025). Here, we have found evidence of population differentiation, albeit concentrated in few genomic areas, between two geographically broad genetic groups. The concentrated nature of population differentiation raises several questions for the management of Northern abalone: how pervasive does the genome differentiation need to be for two groups of individuals to be considered distinct and thus managed as such? And, do those differentiated genomic regions have phenotypic and/or adaptive significance? An increasing number of studies are showing that differentiation can be extremely concentrated in the genome even among what are considered reproductively isolated species (Campagna et al. 2017; Toews et al. 2016), and in many cases a link between these genomic regions and the factors leading to reproductive or ecological isolation between taxa, including populations, ecotypes, or species, has been demonstrated (Barrett et al. 2019; Loveland et al. 2025; Turbek et al. 2021). In Northern abalone, differentiation was mostly associated with genes related to immune response and reproductive functions, but their relative fitness effects are unknown. Understanding if and how these genes affect phenotypes and fitness would require functional validation of different variants or haplotypes and assessment of their fitness effect, but this research is hampered by their relatively long generation times, poor genomic resources for the species (and mollusks more broadly), and their conservation status limiting the necessary experiments. This said, our analyses show that, despite overall low genomic differentiation, the Northern abalone do not represent a single panmictic demographic unit, and that current gene flow among our sampling locations is lower than previously argued (Withler et al. 2003; Dimond et al. 2024). Finally, our demographic reconstructions support existing evidence of a severe population decline and a modest population recovery, at least across the northern part of the Northern abalone range (BC and SE Alaska), which suggests that fishery closures have been effective in slowing the decline and promoting recovery. However, the examined locations are still far from pre‐bottleneck abundances, and continued conservation efforts are crucial to enable further recovery and the long‐term persistence of the species.

## Funding

Whole genome resequencing was funded by the Species At Risk Program (Government of Canada) granted to D.B. GenARCC (GRDI ‐ Government of Canada) provided computational resources.

## Disclosure

Benefit‐Sharing Statement: Sampling was performed under Species at Risk Act (SARA) permits and the coordinates of the sampling sites are not disclosed in accordance with SARA legislation for endangered species. Benefits generated: the results of this research have been shared with Indigenous communities and Fisheries and Oceans personnel to inform their management and conservation. The Committee on the Status of Endangered Wildlife in Canada (COSEWIC) will review the delineation of Designatable Units in Canada based on this research.

## Conflicts of Interest

The authors declare no conflicts of interest.

## Supporting information


**Figure S1:** Manhattan plots of Tajima's *D* values estimated in 50 kb moving windows in the southeast group (upper panel) and in the northwest group (lower panel).
**Figure S2:** Plot of PCA based on 10 highly differentiated SNPs. VI = Vancouver Island, CC = Central Coast, HG = Haida Gwaii, and SEAK = Southeastern Alaska.
**Figure S3:** Plot summarizing demographic reconstructions for each genetic group (NW = northwest, SE = southeast) using three different recombination rates.
**Figure S4:** Boxplot showing the distribution of singletons/doubletons, variants occurring in only one individual as either homozygous or heterozygous, across sampling locations.
**Figure S5:** Boxplots showing the distribution of number (NROH) and length (SROH) of ROHs across sampling locations.
**Figure S6:** Heatmap showing the degree of overlap of ROHs from different sampling locations.
**Table S1:** List of genes affected by the 1000 most differentiated SNPs.

## Data Availability

Raw hole genome resequencing data are available on ENA/NCBI under Project PRJEB112870.
