# Supplementary material for: Whole Genome Analyses of the Endangered Northern Abalone (Haliotis kamtschatkana) Reveal Population Differentiation and a Genomic Signature of a Dramatic Population Decline
Source: Evol Appl. 2026 Jun 4;19(6):e70255. doi: 10.1111/eva.70255 (PMC13238616; doi:10.1111/eva.70255)

**Supplementary material**

Supplementary Table 1. List of genes affected by the 1,000 most differentiated SNPs.

| Original scaffold | Renamed scaffold | Genome annotation | Gene symbol |
| --- | --- | --- | --- |
| NW_025963779.1 | 1 | LOC124131698 | *CLCA4* |
| NW_025963779.1 | 1 | LOC124136951 | *acantho2* |
| NW_025963890.1 | 2 | LOC124114122 | *NCS2* |
| NW_025963890.1 | 2 | LOC124136239 | *MP2K1* |
| NW_025963890.1 | 2 | LOC124153060 | *uncharacterized* |
| NW_025963890.1 | 2 | LOC124134541 | *PTPRE* |
| NW_025964001.1 | 3 | LOC124146455 | *UBR7* |
| NW_025964001.1 | 3 | LOC124119068 | *4CLL* |
| NW_025964001.1 | 3 | LOC124119052 | *CCDC85C* |
| NW_025964001.1 | 3 | LOC124119042 | *TMEM179* |
| NW_025964001.1 | 3 | LOC124119021 | *PRPF39* |
| NW_025964001.1 | 3 | LOC124119001 | *DMAC2L* |
| NW_025964001.1 | 3 | LOC124119019 | *DMAC2L* |
| NW_025964001.1 | 3 | LOC124118992 | *AHSA1* |
| NW_025964001.1 | 3 | LOC124118980 | *USP34* |
| NW_025964001.1 | 3 | LOC124116155 | *uncharacterized* |
| NW_025964112.1 | 4 | LOC124150370 | *PTPRA* |
| NW_025964112.1 | 4 | LOC124132118 | *uncharacterized* |
| NW_025964112.1 | 4 | LOC124132122 | *ZNF91* |
| NW_025964112.1 | 4 | LOC124132123 | *uncharacterized* |
| NW_025964112.1 | 4 | LOC124132126 | *uncharacterized* |
| NW_025964112.1 | 4 | LOC124135902 | *NCAPG2* |
| NW_025964112.1 | 4 | LOC124152397 | *PAQR5* |
| NW_025964112.1 | 4 | LOC124152378 | *CUBN* |
| NW_025964112.1 | 4 | LOC124152367 | *SPAG16* |
| NW_025964112.1 | 4 | LOC124152351 | *CDK5RAP3* |
| NW_025964112.1 | 4 | LOC124152357 | *MANBA* |
| NW_025964334.1 | 6 | LOC125374871 | *HRG* |
| NW_025964361.1 | 7 | LOC124132663 | *PCDH11X* |
| NW_025964372.1 | 8 | LOC124112318 | *AWH* |
| NW_025964372.1 | 8 | LOC124144804 | *PPT2A* |
| NW_025964372.1 | 8 | LOC124142829 | *FLOT1* |
| NW_025964372.1 | 8 | LOC125377020 | *MUC2* |
| NW_025964372.1 | 8 | LOC124138381 | *WDR38* |
| NW_025964372.1 | 8 | LOC124138433 | *RALB* |
| NW_025964372.1 | 8 | LOC124143576 | *uncharacterized* |
| NW_025964383.1 | 9 | LOC124139531 | *uncharacterized* |
| NW_025964383.1 | 9 | LOC124139582 | *uncharacterized* |
| NW_025964383.1 | 9 | LOC124139533 | *MINDY4B* |
| NW_025964383.1 | 9 | LOC124139535 | *PPM1L* |
| NW_025964383.1 | 9 | LOC124139536 | *CUL3* |
| NW_025964383.1 | 9 | LOC124124991 | *uncharacterized* |
| NW_025964383.1 | 9 | LOC124113164 | *uncharacterized* |
| NW_025964383.1 | 9 | LOC125377405 | *uncharacterized* |
| NW_025964383.1 | 9 | LOC124139539 | *TRIP12* |
| NW_025964383.1 | 9 | LOC124139541 | *SERPINB1* |
| NW_025964383.1 | 9 | LOC124128830 | *uncharacterized* |
| NW_025964383.1 | 9 | LOC124139542 | *SELENOT* |
| NW_025964383.1 | 9 | LOC124128829 | *TMEM126A* |
| NW_025964383.1 | 9 | LOC124139544 | *DLG2* |
| NW_025964383.1 | 9 | LOC124145153 | *uncharacterized* |
| NW_025964383.1 | 9 | LOC125377570 | *CPAMD8* |
| NW_025963780.1 | 10 | LOC124143092 | *RPP25L* |
| NW_025963780.1 | 10 | LOC124130871 | *Rya-R* |
| NW_025963791.1 | 11 | LOC124115716 | *RAP2C* |
| NW_025963813.1 | 13 | LOC124115480 | *MFAP4* |
| NW_025963813.1 | 13 | LOC124115479 | *ZNF93* |
| NW_025963813.1 | 13 | LOC124115477 | *uncharacterized* |
| NW_025963813.1 | 13 | LOC125378832 | *uncharacterized* |
| NW_025963813.1 | 13 | LOC124115473 | *DDOST* |
| NW_025963813.1 | 13 | LOC124115472 | *UHRF1* |
| NW_025963813.1 | 13 | LOC124115495 | *uncharacterized* |
| NW_025963813.1 | 13 | LOC124115494 | *uncharacterized* |
| NW_025963813.1 | 13 | LOC124115493 | *slc17.2* |
| NW_025963813.1 | 13 | LOC124111208 | *FucT* |
| NW_025963824.1 | 14 | LOC124118886 | *uncharacterized* |
| NW_025963824.1 | 14 | LOC124113176 | *ANPEP* |
| NW_025963835.1 | 15 | LOC124150975 | *SULT1C2* |
| NW_025963835.1 | 15 | LOC124151000 | *uncharacterized* |
| NW_025963846.1 | 16 | LOC125380946 | *HRG* |
| NW_025963846.1 | 16 | LOC124122464 | *BCAP31* |
| NW_025963868.1 | 18 | LOC124149680 | *NCAM* |
| NW_025963868.1 | 18 | LOC125381607 | *uncharacterized* |
| NW_025963868.1 | 18 | LOC124147989 | *uncharacterized* |
| NW_025963891.1 | 20 | LOC124127588 | *XIAP* |
| NW_025963902.1 | 21 | LOC124133411 | *uncharacterized* |
| NW_025963902.1 | 21 | LOC124133410 | *RHO* |
| NW_025963902.1 | 21 | LOC124133407 | *PRPF19* |
| NW_025963924.1 | 23 | LOC124146525 | *perlucin* |
| NW_025963924.1 | 23 | LOC124146505 | *perlucin* |
| NW_025963924.1 | 23 | LOC124146504 | *uncharacterized* |
| NW_025963924.1 | 23 | LOC124151785 | *uncharacterized* |
| NW_025963924.1 | 23 | LOC124151784 | *uncharacterized* |
| NW_025963957.1 | 26 | LOC125371569 | *uncharacterized* |
| NW_025964046.1 | 34 | LOC125372047 | *GRIK5* |
| NW_025964079.1 | 37 | LOC124140444 | *AVL9* |
| NW_025964079.1 | 37 | LOC124140443 | *EXT2* |
| NW_025964190.1 | 47 | LOC124110894 | *BRCC36* |
| NW_025964235.1 | 51 | LOC124140694 | *CHAC2* |
| NW_025964257.1 | 53 | LOC124144133 | *HMCN2* |
| NW_025964257.1 | 53 | LOC124126442 | *NPHS1* |

Supplementary Figure 1. Manhattan plots of Tajima’s D values estimated in 50 kb moving windows in the southeast group (upper panel) and in the northwest group (lower panel).


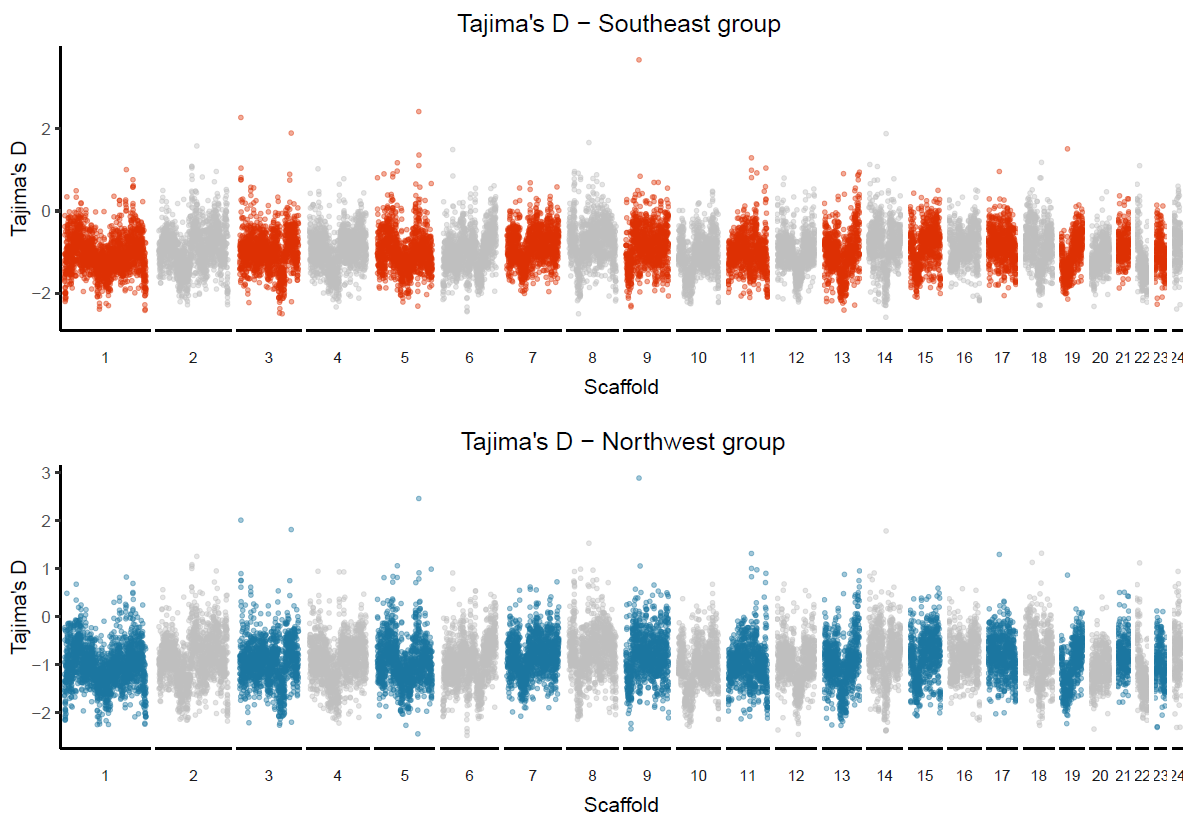


Supplementary Figure 2. Plot of PCA based on 10 highly differentiated SNPs. VI = Vancouver Island, CC = Central Coast, HG = Haida Gwaii, and SEAK = Southeastern Alaska.


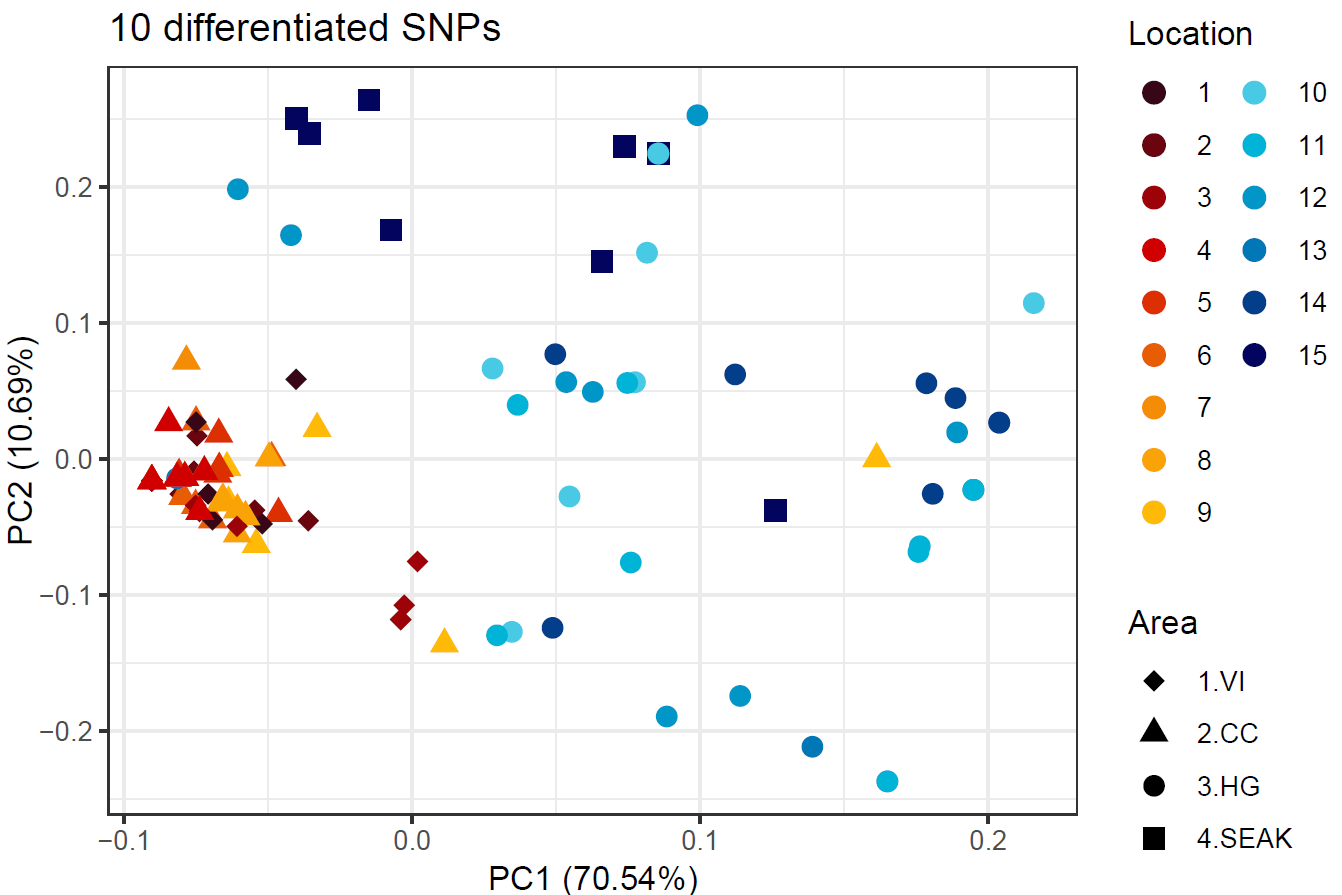


Supplementary Figure 3. Plot summarizing demographic reconstructions for each genetic group (NW = northwest, SE = southeast) using three different recombination rates.


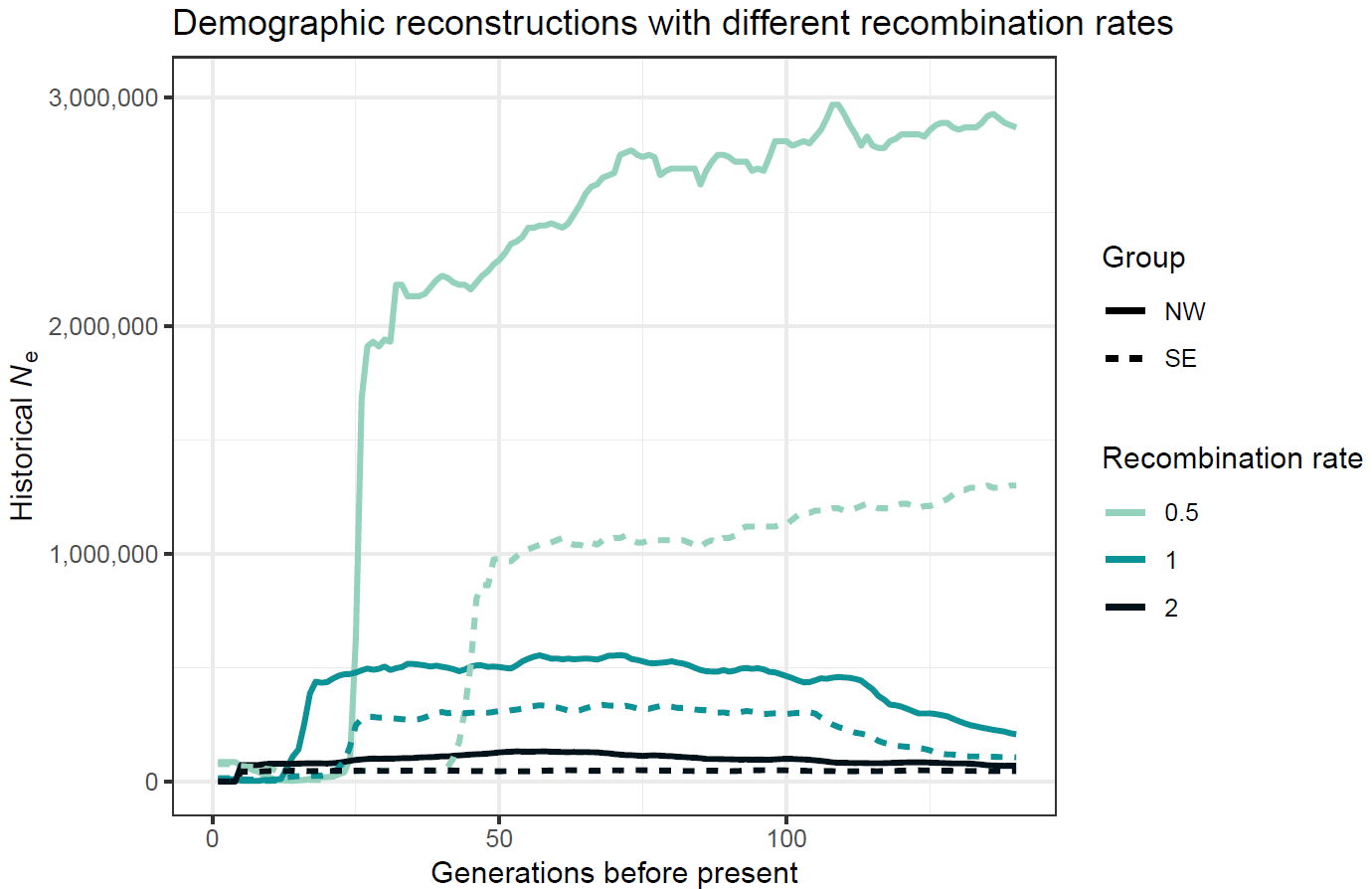


Supplementary Figure 4. Boxplot showing the distribution of singletons/doubletons, variants occurring in only one individual as either homozygous or heterozygous, across sampling locations.


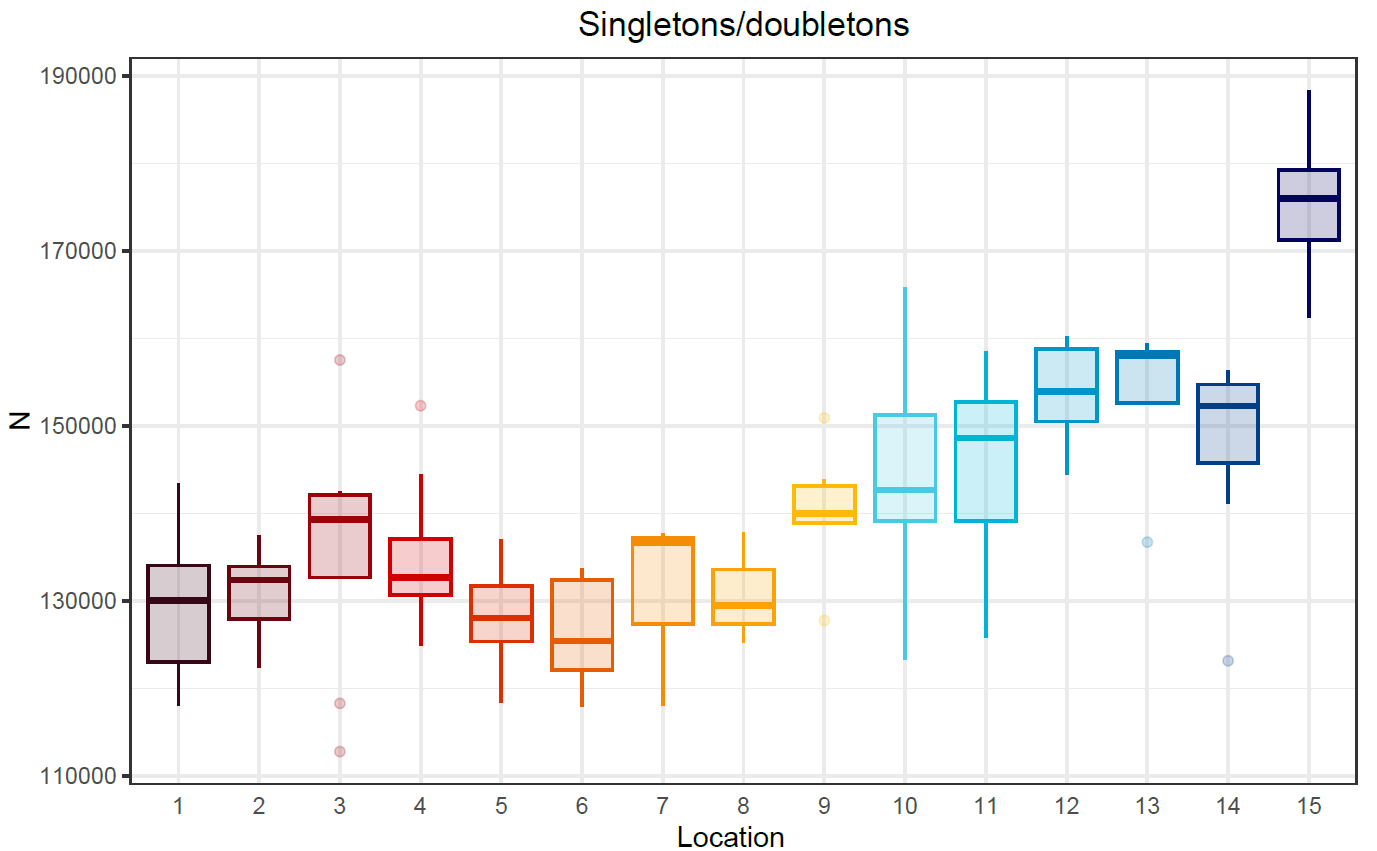


Supplementary Figure 5. Boxplots showing the distribution of number (NROH) and length (SROH) of ROHs across sampling locations.


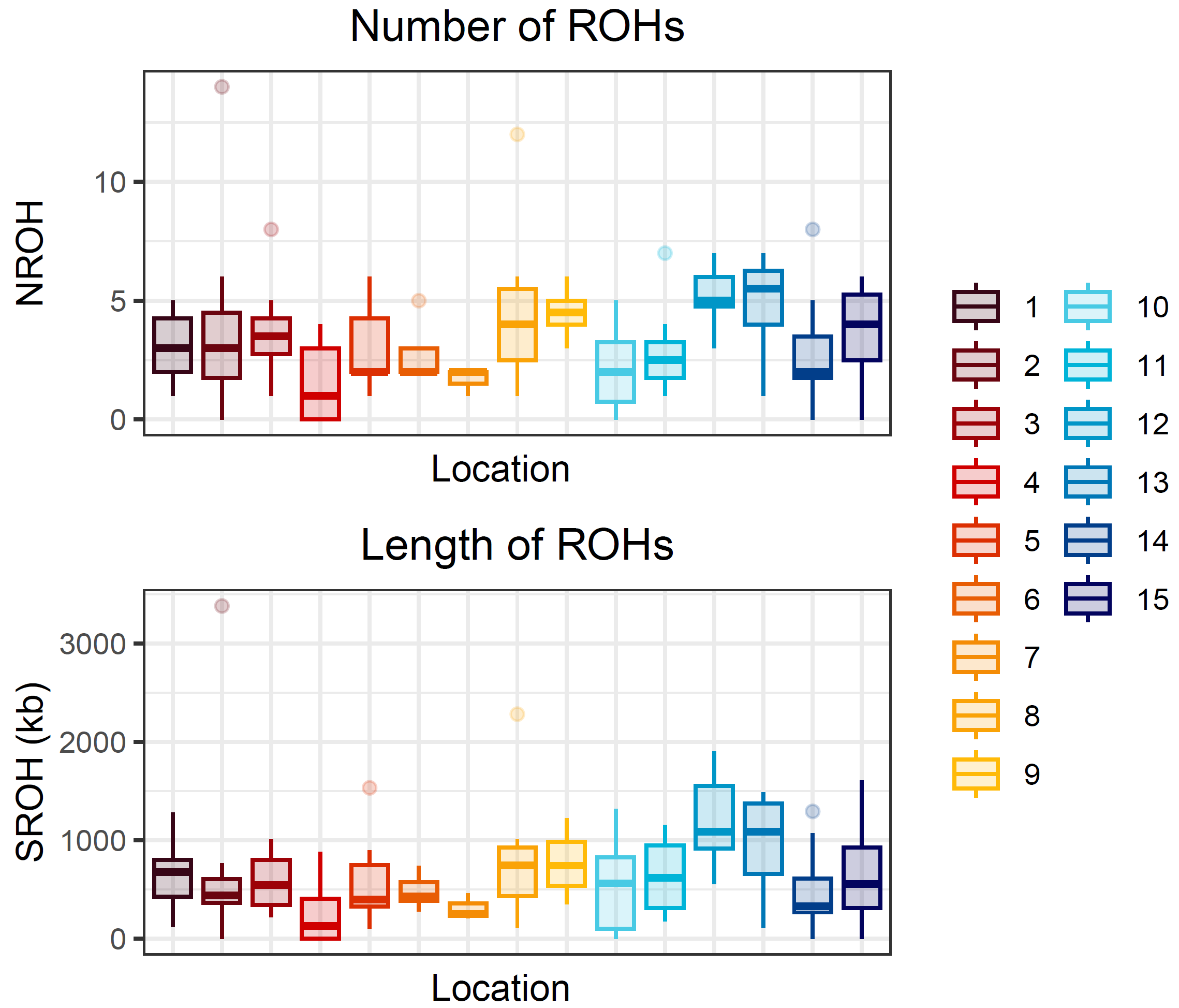


Supplementary Figure 6. Heatmap showing the degree of overlap of ROHs from different sampling locations.


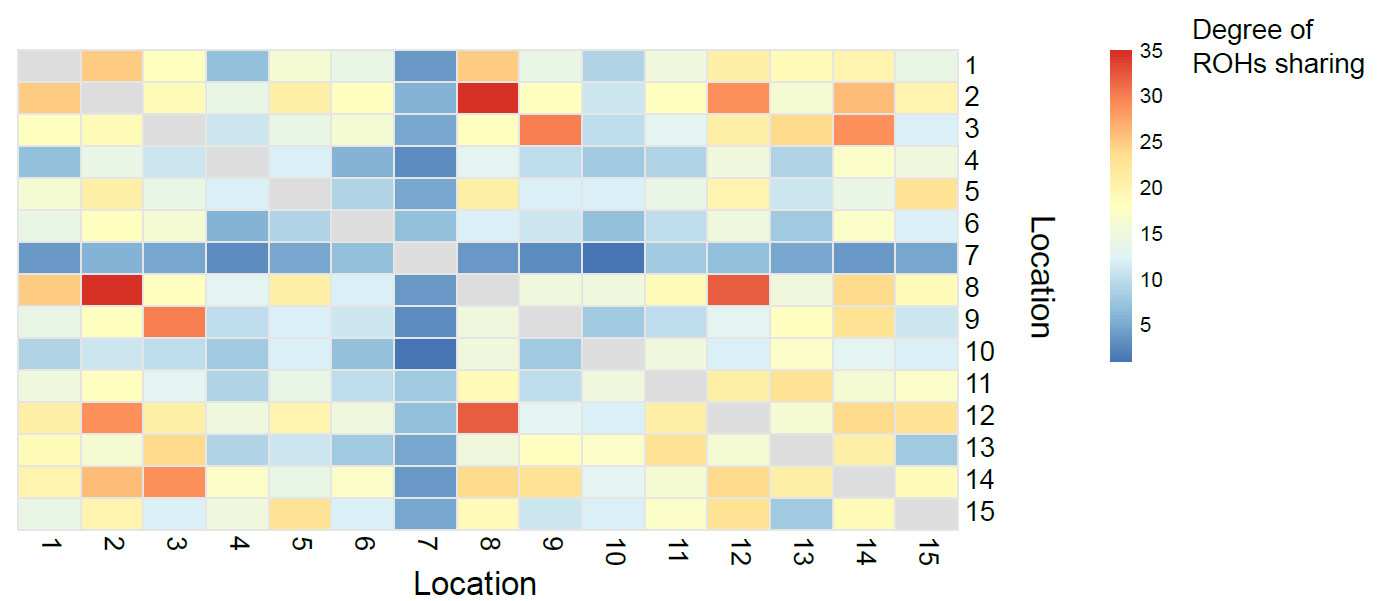

Supplement: Supplementary file 1 — Figure S1: Manhattan plots of Tajima's D values estimated in 50 kb moving windows in the southeast group (upper panel) and in the northwest group (lower panel). Figure S2: Plot of PCA based on 10 highly differentiated SNPs. VI = Vancouver Island, CC = Central Coast, HG = Haida Gwaii, and SEAK = Southeastern Alaska. Figure S3: Plot summarizing demographic reconstructions for each genetic group (NW = northwest, SE = southeast) using three different recombination rates. Figure S4: Boxplot showing the distribution of singletons/doubletons, variants occurring in only one individual as either homozygous or heterozygous, across sampling locations. Figure S5: Boxplots showing the distribution of number (NROH) and length (SROH) of ROHs across sampling locations. Figure S6: Heatmap showing the degree of overlap of ROHs from different sampling locations. Table S1: List of genes affected by the 1000 most differentiated SNPs. [file EVA-19-e70255-s001.docx]
